# Electroacupuncture Treatment Improves Learning-Memory Ability and Brain Glucose Metabolism in a Mouse Model of Alzheimer's Disease: Using Morris Water Maze and Micro-PET

**DOI:** 10.1155/2015/142129

**Published:** 2015-03-03

**Authors:** Jing Jiang, Kai Gao, Yuan Zhou, Anping Xu, Suhua Shi, Gang Liu, Zhigang Li

**Affiliations:** ^1^Beijing University of Traditional Chinese Medicine, Beijing 100029, China; ^2^Institute of Laboratory Animal Science, Chinese Academy of Medical Sciences & Comparative Medical Center, Peking Union Medical College, Beijing 100021, China; ^3^Community Health Service Center of Dongcheng District, Beijing 100010, China

## Abstract

*Introduction*. Alzheimer's disease (AD) causes progressive hippocampus dysfunctions leading to the impairment of learning and memory ability and low level of uptake rate of glucose in hippocampus. What is more, there is no effective treatment for AD. In this study, we evaluated the beneficial and protective effects of electroacupuncture in senescence-accelerated mouse prone 8 (SAMP8). *Method*. In the electroacupuncture paradigm, electroacupuncture treatment was performed once a day for 15 days on 7.5-month-old SAMP8 male mice. In the normal control paradigm and AD control group, 7.5-month-old SAMR1 male mice and SAMP8 male mice were grabbed and bandaged while electroacupuncture group therapy, in order to ensure the same treatment conditions, once a day, 15 days. *Results*. From the Morris water maze (MWM) test, we found that the treatment of electroacupuncture can improve the spatial learning and memory ability of SAMP8 mouse, and from the micro-PET test, we proved that after the electroacupuncture treatment the level of uptake rate of glucose in hippocampus was higher than normal control group. *Conclusion*. These results suggest that the treatment of electroacupuncture may provide a viable treatment option for AD.

## 1. Introduction

Alzheimer's disease (AD) is a progressive neurodegenerative disease, which is the most widespread cause of dementia and its incidence will continue to increase rapidly as the population ages [1]. It is characterized by the progressive decline of memory and cognitive function and changes in behavior and personality [2]. Despite the fact that extensive research is focused on AD, there is no effective treatment for this disease [3]. Therefore, the therapeutic of AD is urgent to be proposed.

Electroacupuncture (EA) treatment is a type of needling therapy from* Journal of Acupuncture and Moxibustion*, number 1,1934, which combines needling with electric stimulation, connecting needles of the point group concerned (2 points make up a group) with pulse current from the electric stimulator [4]. There is a dual-directional pulse current (intermittent oscillatory current), sin wave, square wave, and so forth, with characteristics such as continuous wave, sparse-dense wave, intermittent wave, undulate wave, and saw tooth wave. The frequency most commonly used is 1–1000 times/sec. over 1000 times/sec. which is used less. What is more, the proper intensity of the stimulation is marked by a muscular twitch around the acupoint and a comfortable sensation [5]. EA treatment has advantages like many kinds of oscillation waves, wide range of frequency, and stable function [6].

So far, EA treatment has yielded neuroprotective function in animal models of depression, spinal cord injury [7, 8], cerebral ischemia-reperfusion injury [9–12], stork [9, 10, 12, 13], and many kinds of pain [14–20]. Clinically, EA treatment has been shown to have efficacy in curing many kinds of neurological disease, such as depression [21, 22], spinal cord injury [23–25], cerebral ischemia-reperfusion injury [26], stork [27–29], and many kinds of pain [30–36]. Thus, some of researcher proposed that since the EA treatment could protect the central nervous system it may be used as an alternative treatment for AD [37]. However, more research is needed to prove this conclusion.

The aim of this study was to assess the efficacy of EA treatment in curing AD. We utilized the mouse model of AD, senescence-accelerated mouse prone 8 (SAMP8), which develops the learning and memory impairment and the mood disorder. Here, we present the ethology and in vivo imaging evidence that EA treatment over a period of half a month improves the learning and memory ability and brain glucose metabolism in AD, specifically in the hippocampus of the SAMP8 mouse. This finding extends our previous EA work in the models of the central nervous injury to demonstrate that EA treatment is also effective in protecting the brain against chronic insults due to AD-related disease.

## 2. Method and Materials

### 2.1. Animals

Senescence-accelerated mouse prone 8 (SAMP8) and the cognate normal senescence-accelerated mouse-R1 (SAMR1) breeding pairs were kindly provided by Professor Takeda at Kyoto University, Japan [38]. The animals were housed in a barrier facility of the* Experimental Animal Centre of First Teaching Hospital of Beijing University of Traditional Chinese Medicine* and under live conditions of controlled temperature (24 ± 2°C), a 12 h/12 h dark/light cycle, and sterile drinking water and standard pellet diet ad libitum. All experiments were performed according to the National Institute of Health Guide for the Care and Use of Laboratory Animals (NIH publications number 80-23). Thirty 7.5-month-old SAMP8 male mice were divided into two groups (*n* = 10 per group): SAMR1 normal control (Rc) group, SAMP8 Alzheimer's disease control (ADc) group, and SAMP8 electroacupuncture (EA) group.

### 2.2. Acupuncture Manipulation

In the EA group, electroacupuncture treatment was performed once a day for 15 days (no treatment on the eight day). The prescription of acupuncture points included DU20* Baihui*, DU 26* Shuigou,* and EX-HN3* Yintang* (the significant extra point). The locations of these points were according to the* National Acupuncture Society for Experimental Research* developed the “laboratory animal acupuncture atlas”.* Huatuo* card 30#, 0.5 inch needle was used for treatment. Pricking method was used for DU 26* Shuigou*; flat thorn method was used for DU20* Baihui* and EX-HN3* Yintang*. Needle depth was 0.5 cm and taped. The needle handle was connected with HANS-LH202 electroacupuncture device (*Peking University Institute of Science Nerve and Beijing Hua Wei Industrial Development Company*), sparse wave, 2 Hz of the frequency, 2 V of the voltage, and 0.6 mA of the current intensity.

In the Rc group and ADc group, do not do any treatment under the same rearing conditions, while grabbing and bondage the mice in order to ensure the same treatment conditions, once a day, 15 days.

### 2.3. Morris Water Maze Behavioral Test

The Morris water maze consisted of a circular tank (90 cm in diameter, 50 cm in height) filled with water to a depth of 29 cm maintained at 24 ± 1°C and rendered opaque with blue-black ink. A removable circular platform (9.5 cm diameter, 28 cm height) with its top surface 1 cm below the water was located inside the pool. The area of the pool was conceptually divided into four quadrants (NE, NW, SW, and SE) of equal size. Data were collected by a video camera (TOTA-450*Ш*, Japan) which was fixed to the ceiling of the room and connected to a video recorder and an automated tracking system (China Daheng Group, Beijing, China).

In this behavioral test, mice are placed in the pool of water containing a platform just below the surface of the water. They escape from the maze when they find the platform. Distal visual cues are arrayed around the room, and in general, mice are able to learn the location of the hidden platform based on these cues.

### 2.4. Hidden Platform (Place) Testing

This portion of the test assesses the ability of the mice to find the platform under conditions where they cannot directly see it but must either remember it is relative to external cues or perform a search for it. The platform was placed 1 cm under the surface of the water, and the water was opaque by a suspension of dark blue, nontoxic tempera paint. The platform was placed in a different location from that used in the visible platform testing. Each mouse was released from one of 4 locations and had 60 s to search for the hidden platform. At the end of each trail, the mouse was placed on the platform or allowed to stay on the platform for 15 s. Prominent spatial cues were arrayed around the room. The investigator is also a powerful spatial cue and always sat in the same location during each trail after releasing the mouse. Eight trails per day for 4 consecutive days were performed with the location of the platform kept constant. We recorded the time that the mouse found the platform needed, and we call it escape latency.

### 2.5. Probe Trail

The day after the completion of hidden platform testing, the platform was removed, and each mouse was placed in the pool once for 60 s, starting from the same starting location as was used first in hidden platform testing. The time spent swimming in the quadrant where the platform had been was recorded. This is considered to be the most specific test for spatial memory. We recorded the time that the mouse spent in the platform quadrant and calculated the percentage of total time spent in swimming to the platform quadrant.

### 2.6. Micropositron Emission Tomography

Before experiments, each mouse (7.5 months, 28~32 g) for blood glucose monitoring, the results showed the normal range (7.0~10.1 mmol/L) could be used for micro-PET detective (^18^F-FDG PET tracer was provided by the Chinese Medicine Research Institute PET Room; PET imaging system using Siemens INVEON PET/CT imaging system). Six hours of water deprivation before the experiment. The mice were placed in the suction chamber, inhaling the oxygen mixed with 1.5% isoflurane to be anesthetized. After complete anesthesia, approximately 14.8~16.5 MBq ^18^F-FDG PET tracers were injected via vena of tail. After the ^18^F-FDG PET tracer uptake for 60 min, the mice were placed on the scan bed in prone position, the mice and scanner long axis were parallel, and the head of mouse was located within the scanner field of view. Then the micropositron emission tomography began to collect the image. During this progress, the mice were anesthetized by the oxygen mixed with 1.5% isoflurane (1 L/min).

### 2.7. Micropositron Emission Tomography Image Reconstruction

Filtered back projection (FBP) and CT photon attenuation correction were used for image reconstruction. Dynamic micro-PET image frames are taken 30 s/frames.

### 2.8. Region of Interest Selection

The three-dimensional region of interest technology was applied for manual selection of the hippocampus three-dimensional region of interest in transverse, coronal, and sagittal planes. Then calculate the uptake rate of per gram with the region of interest.

### 2.9. Statistical Method

All data were analyzed by SPSS (version 17.0; SPSS, Inc., Chicago, IL, USA). All measurements were performed by an independent investigator blinded to the experimental conditions. The results in the figures are expressed as the mean ± standard deviation. Differences within or between normal distributed data were analyzed by analysis of variance (ANOVA) followed by Huynh-Feldt test (for Morris water maze test). Statistical significance was set at *P* < 0.05.

## 3. Results

### 3.1. Effect of Electroacupuncture in Spatial Learning Ability of SAMP8 Mouse in the Morris Water Maze Test

**Figure 1:**
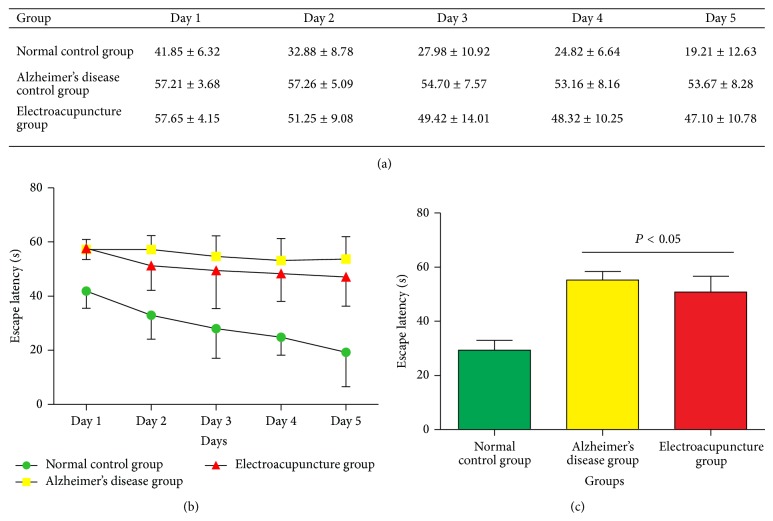
(a) Comparison of the escape latency of all groups. (b) The trend of the escape latency of all groups. (c) Comparison the mean of the escape latency of all groups.

The effect of electroacupuncture in spatial location ability of SAMP8 mouse in the WMW test is elucidated in Figure 1(a). We can see that with the training time extension, the escape latency of all groups had shown a downward trend (Figure 1(b)). The AD control group showed marked retardation in the escape latency, probably due to the memory deficits resulting from the rapid aging process impairment of learning and memory. The analysis of the escape latency revealed that the mouse in EA group had significantly reduced the escape latency compared with the AD control group (*P* < 0.05, Figure 1(c)).

### 3.2. Effect of Electroacupuncture in Spatial Memory Ability of SAMP8 Mouse in the Morris Water Maze Test

**Figure 2:**
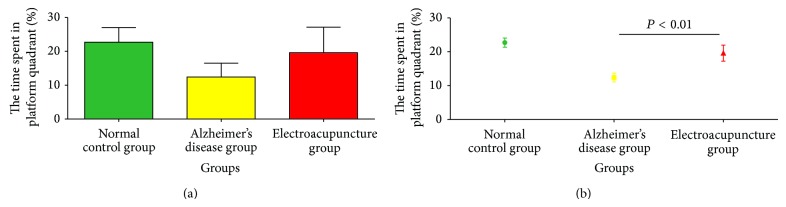
(a) The percentage of time spent in platform quadrant of each group. (b) The percentage of time spent in platform quadrant of each group.

To investigate the effect on spatial memory ability, the performance in the probe trial on day 6 was examined by analyzing the percentages of time spent swimming to the expected position of the platform. A higher percentage of time spent in the platform quadrant is interpreted as a higher level of memory retention [39]. In this probe trial, we found that compared with AD control group, EA group spent higher time in the platform quadrant (*P* < 0.01). What is more, in percentage of time spent in the platform quadrant, EA group and normal group had no significant difference in statistics (*P* = 0.223, Figure 2).

### 3.3. PET Imaging of Mice Hippocampus

Because of the effects of the tail vein injection, condition of anesthesia, and the metabolism of the ^18^F-FDG, four animals of each group could successfully guarantee the completion of micro-PET test.

**Figure 3:**
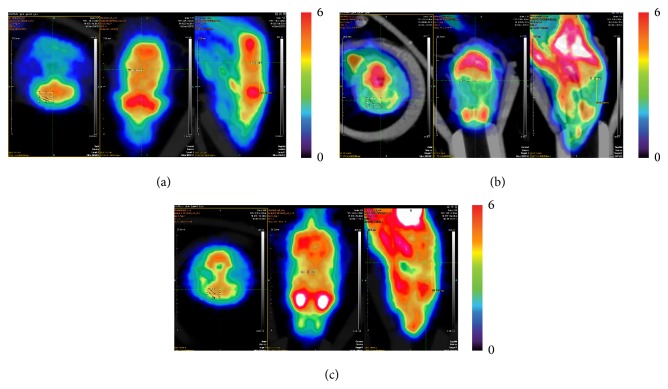
Right side hippocampus of the mice in micro-PET scan image. (a) Normal control group; (b) Alzheimer's disease group; (c) electroacupuncture treatment group. Color code: min = 0, max = 6.

Use the same color standard and color code from top high to the bottom low to display the metabolic rate of the glucose. The left of the observer is the right of the animal. From the image, after the treatment of electroacupuncture the ^18^F-FDG of hippocampus is higher than Alzheimer's disease group (Figure 3).

### 3.4. ^18^F-FDG Uptake Rate of per Gram in Hippocampus Tissue

**Figure 4:**
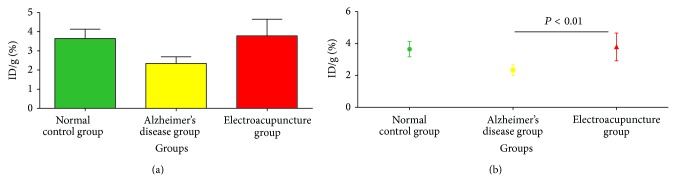
(a) The uptake rate of ^18^F-FDG per gram in hippocampus. (b) The uptake rate of ^18^F-FDG per gram in hippocampus.

To study that the treatment of electroacupuncture corresponds to enhancing the glucose metabolic activity in hippocampus, ^18^F-FDG PET scan was performed on the mice. The result showed that after treatment of electroacupuncture the uptake rate of ^18^F-FDG in hippocampus was higher than Alzheimer's disease group and normal control group (Figure 4).

## 4. Discussion

In the current research, we studied the effect of electroacupuncture on animal model of Alzheimer's disease using Morris water maze and micro-PET and aimed to find that whether the treatment of electroacupuncture can improve the condition of Alzheimer's disease. Using the Morris water maze, we found after the treatment of electroacupuncture that the spatial learning and memory ability of the SAMP8 mouse had improved. Further, the result of micro-PET revealed that treatment of electroacupuncture can increase the uptake rate of glucose in hippocampus of SAMP8 mouse. These findings from animal behavior and in vivo imaging lead us to conclude that the treatment of electroacupuncture may play a curable role in Alzheimer's disease, particularly in the learning and memory ability.

### 4.1. Alzheimer's Disease in Traditional Chinese Medicine and the Acupuncture Prescription

Alzheimer's disease belonged to encephalopathy in Chinese medicine. It is caused by deficiency of* jing and blood*, with the aged condition, leading to serious brain function disorder. It is characterized by forgetful and personality changes [40]. In the treating principle of Chinese medicine, according to the principle “the brain is the house of mentality,” “the Governor Vessel … entering the brain and extending up to the very top of the head,” based on the close relationship brain-mentality and brain-Governor Vessel, we proposed “dredging the Governor Vessel and awakening mind” method to treat Alzheimer's disease.

In the selection of acupoints, we chose DU20* Baihui*, DU 26* Shuigou,* and EX-HN3* Yintang* as the main points. DU20* Baihui*, a meridian point of the Governor Vessel, and the meeting point of Governor Vessel, the three Yang Meridians of the hand and foot, from the* A-B Classic of Acupuncture and Moxibustion* (*zhen jiu jia yi jing*), also named* Sanyangwuhui*,* Dianshang*,* Wuhui*. It is located on the head, 5 cun directly above the midpoint of the anterior hairline. Its indication is headache, dizziness, palpitation due to fright, amnesia, corpse-like syncope, aphasia from apoplexy, manic-depressive psychosis, epilepsy, hysteria, and so forth. DU 26* Shuigou* is a point on the Governor Vessel, Hand-Yangming, and Foot-Yangming, also called* Renzhong*. Its location is on the face, at the junction of the upper 1/3 and middle 1/3 of the philtrum. It is used for coma, syncope, manic-depressive disorder, epilepsy, acute and chronic infantile convulsion, and so forth. EX-HN3* Yintang*, an extra point, is seen in* Bian Que's Jade Dragon Classics of Acupuncture and Moxibustion* (*Bian Que Shenying Zhenjiu Yulong Jing*). It is on the forehead, at the midpoint between the eyebrows. Headache, vertigo, insomnia, and puerperal faintness are the indication of EX-HN3. In general, we use the above three acupoints as the acupuncture prescription.

### 4.2. Senescence-Accelerated Mouse-P8 (SAMP8) Is an Optional Animal Model for Alzheimer's Disease

The senescence-accelerated mouse (SAMP8) is a spontaneous animal model of Alzheimer's disease, and it develops early memory disturbances and changes in the blood-brain barrier resulting in decreased efflux of amyloid-beta protein from the brain [38]. This nontransgenic animal model with great utility can be better simulated for the memory deficits and the low level uptake rate of glucose in hippocampus [41]. So, in the current research, this kind of animal model can help us to find the effect of electroacupuncture treatment in curing Alzheimer's disease.

### 4.3. Effect of Electroacupuncture in Morris Water Maze

The Morris water maze (MWM) is one of the most common tasks used to assess spatial learning and memory ability in rodents [42]. Spatial navigation performance in the hidden goal task (HGT), a real-space human analogue of the Morris water maze, can identify mild cognitive impairment (MCI) patient with memory impairment of the hippocampus type, a known indicator of incipient Alzheimer's disease [43]. In our study, we found that after the treatment of electroacupuncture, the spatial learning and memory ability of the SAMP8 mouse had improved compared with the nontreatment SAMP8 mouse, which suggested that the electroacupuncture may improve the cognitive ability of Alzheimer's disease patients.

### 4.4. Effect of Electroacupuncture in Micro-PET

Studies suggested that the cognitive impairment of Alzheimer's disease to a certain extent results from the low level of uptake rate of glucose in hippocampus [44]. So in our current study, we used the micro-PET to get the in vivo image of the uptake rate of glucose in hippocampus.

Positron emission tomography is a noninvasive functional brain imaging technique at the molecular level, which makes the use of radioactive marker to analyze the metabolism condition in the brain, images the distribution of biologically targeted radiotracer with high sensitivity [45]. It can directly reflect the activity of neurons, which becomes an important tool for diagnosing disease and evaluating efficacy [46]. With the growing importance of animal research in modern molecular biology, the appearance of micropositron emission tomography (micro-PET) makes the possible of in vivo molecular imaging. Development of micro-PET instrumentation for small animal imaging and the availability of positron-emitting tracers have made this technology accessible for the noninvasive, quantitative, and repetitive imaging of biological function in living animals. The development of new probes and positron-imaging based reporter genes has extended micro-PET applications to investigations of metabolism, enzyme activity, receptor-ligand interactions, protein-protein interactions, gene expression, adoptive cell therapy, and somatic gene therapy [47].

In this research, the ^18^F-FDG uptake condition in the hippocampus of mice was imaged by micro-PET, which can show the metabolism level in the hippocampus of Alzheimer's disease mice. Seeing from the images, the ^18^F-FDG uptake condition of electroacupuncture treatment group is higher than Alzheimer's disease group. With further calculation and comparison of the ^18^F-FDG uptake rate of each group, we can see that the electroacupuncture treatment group is the highest, normal control group is in the middle, and Alzheimer's disease group is the lowest. The above results showed, after the electroacupuncture treatment the glucose metabolism level in the hippocampus of the Alzheimer's disease animal model would be higher. Therefore, we could draw the conclusion that the treatment of electroacupuncture could improve the level of uptake rate of glucose in hippocampus in Alzheimer's disease animal.

## 5. Conclusion

In this research, using the test of Morris water maze and the micro-PET in Alzheimer's disease animal model SAMP8 mouse, we found that the treatment of electroacupuncture can improve the spatial learning and memory ability by heightening the level of uptake rate of glucose in hippocampus. This is an interesting notion; however, further research is needed to prove.
